# The Association of Air Pollution Exposure With Glucose and Lipid Levels: The Role of an Extreme Air Pollution Event Alongside 2 Decades of Moderate Exposure

**DOI:** 10.1093/aje/kwad173

**Published:** 2023-08-16

**Authors:** Pablo Knobel, Allan C Just, Elena Colicino, Susan L Teitelbaum, Mary Ann McLaughlin, Heresh Amini, Maayan Yitshak Sade

**Keywords:** cholesterol, diabetes, extreme air pollution events, glucose, particulate matter, World Trade Center

## Abstract

Extreme air pollution events and moderate exposure to fine particulate matter (PM_2.5_) are associated with increased cardiometabolic risk. The World Trade Center (WTC) Health Program general responder cohort includes responders to the WTC disaster. We investigated whether their exposure to this extreme air pollution event (2001) was associated with long-term metabolic outcomes, independently from the associations of intermediate-term PM_2.5_ exposure later in life (2004–2019). We included 22,447 cohort members with cholesterol (*n* = 96,155) and glucose (*n* = 81,599) laboratory results. Self-reported WTC exposure was derived from a questionnaire. PM_2.5_ exposure was derived from a satellite-based model. We observed an increase of 0.78 mg/dL (95% confidence interval (CI): 0.30, 1.26) in glucose and 0.67 mg/dL (95% CI: 1.00, 2.35) in cholesterol levels associated with an interquartile range increase in PM_2.5_ averaged 6 months before the study visit. Higher WTC-exposure categories were also associated with higher cholesterol (0.99 mg/dL, 95% CI: 0.30, 1.67, for intermediate exposure) and glucose (0.82 mg/dL, 95% CI: 0.22, 1.43, for high exposure) levels. Most associations were larger among people with diabetes. Extreme air pollution events and intermediate PM_2.5_ exposure have independent metabolic consequences. These exposures contributed to higher glucose and lipids levels among WTC responders, which may be translated into increased cardiovascular risk.

## Abbreviations

CI: confidence interval
IQR: interquartile range
PM_2.5_: particulate matter with a diameter less or equal to 2.5 μm
WTC: World Trade Center
WTCHP: World Trade Center Health Program

Heart disease is the leading cause of death in the United States (1), and mortality rates remain high despite advancements in prevention and treatment. Therefore, identifying modifiable environmental exposures associated with increased cardiometabolic risk is crucial. Increased exposure to fine particulate matter (particulate matter with a diameter of less or equal to 2.5 μm (PM_2.5_)) has been linked to increased diabetes risk (2–4). PM_2.5_ has also been linked to serum glucose and lipoprotein levels (5, 6)—known predictors of cardiometabolic disease risk (7). Concurrently, studies have showcased a potential effect modification by diabetes status in the associations between air pollution and cardiometabolic health, including more substantial impacts of intermediate-term PM_2.5_ exposure on glucose, low-density lipoprotein, and triglycerides among people with diabetes (6).

Beyond the associations of short (i.e., 1 week) and intermediate-term (i.e., 3 months, 6 months) air pollution exposure, extreme air pollution events have also been associated with a higher risk of congestive heart failure and ischemic heart disease (8). However, no study has evaluated the independent associations of extreme air pollution exposure and subsequent moderate PM_2.5_ exposure with metabolic biomarkers. High glucose levels increase cardiovascular disease risk through cell toxicity and metabolic abnormalities (9). High total cholesterol levels increase vascular damage and induce arteriosclerosis (10). Additionally, both biomarkers are highly correlated with other cardiovascular disease risk factors.

The World Trade Center Health Program (WTCHP) general responder cohort is a unique population exposed to an extreme air pollution event with 2 decades of cohort data available. An unprecedented extreme air pollution event was experienced by over 90,000 people that were involved in the massive rescue, recovery, and clean-up effort after the September 11, 2001, terrorist attack on the World Trade Center (WTC) (11).

Studies investigating the cardiometabolic risk associated with WTC-related exposures found contradicting results (12–14), and none have incorporated the health outcomes associated with PM_2.5_ exposure in the years following the attack. For example, a 2014 study found an increased risk for diabetes following WTC stress-related exposures (12). Others, however, have found lower-than-expected rates of metabolic syndrome (13) and diabetes (14) compared with those in the general population.

Some exposures evaluated in these studies are unique to the WTC tragedy. Dust samples collected after the attack were comprised primarily of elements attributed to collapsed buildings, such as calcium sulfate and calcium carbonate (15), fiberglass, asbestos (15), and lead (16). Although large particles dominated the collapsed buildings’ dust, inhalable particles were suspended in the area for longer. Moreover, the settled WTC dust was unique in its high probability of resuspension, even given minimal air movement (16). Air monitoring data shows that PM_2.5_ levels returned to typical urban New York city levels months after the attack (16). These unique properties of the dust, along with exposures from the burning towers, enhanced and prolonged inhaled exposures by responders beyond the day of the attack. While the dust composition is unique to the 9/11 attack (16), this massive environmental exposure can be conceptually compared with other extreme events featuring high particulate air pollution exposure, like wildfires (8). Quantitative comparisons of dust composition are challenging due to limited data availability; however, the enhanced and prolonged inhaled exposures are comparable. Since populations exposed to wildfire smoke often experience these events repeatedly, the cardiometabolic consequences of wildfire smoke exposure might even be greater. As extreme air pollution exposure events are becoming more common due to climate change (17), understanding their implications on cardiometabolic health is essential.

In this study, we simultaneously assessed the differences in metabolic biomarkers associated with WTC-related exposure (2001) and subsequent short- and intermediate-term PM_2.5_ exposures averaged before the participants’ study visits (2004–2019). Using data from the WTCHP general responder cohort, we assessed whether historical exposure to extreme air pollution events is associated with glucose and total cholesterol levels, independently of the subsequent short- and intermediate-term PM_2.5_ exposure in the 2 decades following the 9/11 attack. We also assessed the differential associations among people with and without diabetes. We hypothesized that both moderate short- to intermediate-term PM_2.5_ exposure and historic WTC-related exposure are independently associated with higher biomarker levels, increasing the risk of cardiometabolic disease among WTC responders.

## METHODS

### Study population

We included repeated measurements of blood glucose (*n* = 82,015) and total cholesterol (*n* = 96,155) obtained from members of the WTCHP cohort (2004–2019). The cohort repository contains 20 years of follow-up data for self-selected workers and volunteers who participated in the clean-up, rescue, and recovery mission after the 9/11 terrorist attack on the WTC towers. Most of the participants were recruited through their participation in earlier programs. The cohort does not include Pentagon, Shanksville, and Fire Department of New York responders. Eligibility criteria are described in detail by Wisnivesky et al. (18). All enrolled participants were administered an exposure assessment questionnaire and a self-administered medical questionnaire on their first monitoring visit. Throughout all monitoring visits, participants were administered an interviewer-administered medical questionnaire and a physical examination, laboratory tests, and pulmonary function tests. The blood glucose and total cholesterol samples were gathered during these monitoring visits. More details on the cohort can be found in Dasaro et al. (11).

We excluded visits of responders residing outside the New York metropolitan area (New York, New Jersey, Connecticut, and Pennsylvania) because, for responders residing in remote states, data regarding the location of the visit was not collected. Therefore, exposure assigned at the residential address might not represent these responders’ short-term air pollution exposure. We excluded cholesterol and glucose values higher than the 99.5th percentile (cholesterol of >323 mg/dL and glucose of >316 mg/dL) to avoid bias due to outcome outliers.

### Exposure assessment

#### Extreme air pollution exposure (2001).

WTC-related exposure information was gathered in the exposure assessment questionnaire during the cohort participants’ first monitoring visit. The questionnaire includes questions about different hazards, including location and length of exposure. All WTC exposure data is self-reported, as no independent exposure measures exist. We used a derived exposure variable that incorporates data on total time working at the WTC site, exposure to the cloud of dust from the WTC collapse, and direct work on the pile of debris. Low exposure included participants who worked less than 40 days, were not exposed to the dust, and did not work directly on the pile. Intermediate exposure includes participants who were not exposed to the cloud but worked 40–90 days or directly on the pile. High exposure includes participants directly exposed to the cloud or working for more than 90 days. We treated these exposure categories as a proxy for the level of air pollution exposure following the 9/11 attack (18). This exposure was used in many other studies in which worse health outcomes were associated with higher WTC-related exposures, suggesting that this variable discriminates well between levels of exposure (18–20).

#### Subsequent PM_2.5_ exposure (2004–2019).

We used a model (21) to estimate residential PM_2.5_ exposure. This model predicts daily mean PM_2.5_ levels on a 1-km resolution. This robust yet parsimonious model uses extreme gradient boosting modeling (XGboost) with satellite-derived aerosol optical depth (AOD) jointly with a recursive feature selection from land-use variables. The model had a root mean square error (RMSE) of 3.11 μg/m^3^ using spatial cross-validation withholding nearby sites and 2.10 μg/m^3^ using random 10-fold data set splitting. More details on the model can be found in Just et al. (21). For visits in or after 2012, we matched exposure to the address at the time of the monitoring visit. Since address history was unavailable for earlier years, we used the first reported address for visits before 2012. We a priori defined the exposure windows as short term (i.e., 1-week average) or intermediate term (i.e., 3- or 6-month average) based on evidence from previous studies (22–25).

### Covariates

We obtained the following covariates from the WTCHP repository: sex, age at the visit, race (non-Hispanic White, non-Hispanic Black, and others), educational level (no high school diploma, high school, some college, and college or other professional schools), diabetes (yes/no), and hypertension (yes/no). Diabetes incidence year was determined based on self-reports, and disease status was assigned for each visit based on diagnosis year. Additionally, we adjusted for residential average temperature matching the exposure window of PM_2.5_. We derived temperature exposure from an XGBoost model to estimate hourly air temperature on a 1-km resolution. More details on the model method and performance can be found in Just et al. (21).

### Statistical analysis

We used generalized additive linear models to estimate the change in glucose and total cholesterol levels associated with a higher WTC-exposure category and 1–interquartile range (IQR) change of PM_2.5_ exposure. Both exposures were included simultaneously in each model to obtain independent associations for each one. We constructed a directed acyclic graph (DAG) based on existing literature to provide a causal framework and select potential confounders (Web Figure 1, available at https://doi.org/10.1093/aje/kwad173). We adjusted our models for sex, age at the study visit, race, education level, diabetes, hypertension, and testing season. We used a penalized spline for the year of testing to control for the time trend. We added a random intercept for each participant to account for potential clustering due to repeated measurements collected over several study visits during the observation period. We fitted a model for each outcome and each of the 3 different PM_2.5_ exposure windows: 1-week, 3-month, and 6-month averages.

#### Secondary statistical analyses.

To evaluate the associations of the exposures with abnormal glucose and total cholesterol levels, we dichotomized the lab results considering glucose of >100 mg/dL and total cholesterol of >200mg/dL at the time of testing as abnormal (26). To assess the role of diabetes as a potential modifier, we stratified our sample by diabetes status at the testing time. Additionally, to assess the potential for reverse causation caused by preexisting conditions during the WTC attack, we assessed the association between diabetes and hypertension diagnosed before 2001 and WTC exposure level. Finally, in the unlikely scenario that higher WTC-related exposure and its associated psychological stress drove study participants to relocate into a rural environment and consequently altered their consequent PM_2.5_ exposure, PM_2.5_ might be considered a mediator on the pathway linking WTC-related exposures and cardiometabolic health. Therefore, we performed mediation analyses to explore the role of PM_2.5_ exposure as a mediator in the association between WTC exposure and both glucose and cholesterol levels. First, we fitted a linear regression adjusted for year and socioeconomic indicators (i.e., race and education level) to test the association between WTC-related exposure and 6-month average PM_2.5_ exposure to determine whether PM_2.5_ is a potential mediator. We selected a 6-month average PM_2.5_ as this was found to be the critical exposure window in our main analysis. Second, we conducted mediation analyses: 1) We fitted mediator models, in which we assess the associations of the WTC-related exposure with 6-month average PM_2.5_; 2) we fitted outcome models to assess the associations of the WTC-related exposure with each outcome including PM_2.5_ in the models; and 3) we combined these outputs and estimated the direct, indirect, and total associations of the WTC-related exposure with glucose or total cholesterol. All models in the mediation analysis adjusted for the same variables as the main analysis. Finally, we repeated the main analysis without adjustment to PM_2.5_ to assess the differences in the effect estimates for the associations between WTC-related exposure and glucose and total cholesterol levels.

#### Sensitivity analysis.

We conducted a sensitivity analysis restricting the data to 2012–2019, when address histories were collected. We repeated the main analysis among the restricted data set to ensure our results were not biased due to measurement error. Finally, we conducted a sensitivity analysis substituting the composite WTC exposure index for the number of hours spent on the WTC site during the clean-up, rescue, and recovery.

## RESULTS

We included 96,155 total cholesterol and 81,599 glucose blood test results drawn between 2004 and 2019 from WTCPH cohort participants (*n* = 22,447). The average age at testing was approximately 52 (the minimum was 21 and the maximum was 92), 85% were male, 62.6% were non-Hispanic White, and 13.1% had diabetes. Glucose and total cholesterol measurements averaged 102.02 mg/dL and 194.80 mg/dL, respectively. In addition, 9.4% of the samples were glucose abnormal (>100 mg/dL), and 11.5% were total cholesterol abnormal (>200 mg/dL). The 6-month average for PM_2.5_ exposure was 8.24 μg/m^3^, and the IQR was 3.09. IQRs for the 3-month and 1-week averages for PM_2.5_ were 3.24 and 4.20, respectively. In addition, 14.5% (13,929) of the participants were in the lowest WTC-exposure category, 63.9% (61,466) were in the intermediate category, and the other 21.6% (20,760) were at the highest exposure category (Table 1). Web Table 1 shows the population characteristics of blood test results excluded from the analytical data set. Excluded test results due to missingness (*n* = 13,559) and included test results had similar characteristics except for a higher percentage of non-Hispanic White individuals and a slightly lower percentage of WTC-related exposure in the included test results (Web Table 1).

**Table 1:** Population Characteristics, World Trade Center Health Program, New York, 2004–2019

		**Diabetes**				
**Characteristic**	**Overall (*n* = 96,155)**		**No (*n* = 83,511)**		**Yes (*n* = 12,644)**	
	**No.**	**%**	**No.**	**%**	**No.**	**%**
Sex, male	81,774	85.0	70,963	85.0	10,811	85.5
Age at testing^a^	51.92 (9.33)		51.16 (9.19)		56.93 (8.70)	
BMI^a^^,^^b^	30.01 (4.94)		29.71 (4.80)		31.98 (5.39)	
Race						
Non-Hispanic White	60,216	62.6	53,681	64.3	6,535	51.7
Non-Hispanic Black	11,525	12	9,284	11.1	2,241	17.7
Other	24,414	25.4	20,546	24.6	3,868	30.6
Educational level						
Less than high school	7,717	8	6,420	7.7	1,297	10.3
High school	20,435	21.3	17,433	20.9	3,002	23.7
Some college	26,746	27.8	23,225	27.8	3,521	27.8
College or professional school	41,257	42.9	36,433	43.6	4,824	38.2
Diabetes	12,644	13.1	0	0	12,644	100
Hypertension	40,897	42.5	31,795	38.1	9,102	72
WTC composite exposure						
Low	13,929	14.5	12,145	14.5	1,784	14.1
Intermediate	61,466	63.9	53,403	63.9	8,063	63.8
High	20,760	21.6	17,963	21.5	2,797	22.1
PM_2.5_, μg/m^3^^a^	8.24 (2.23)		8.31 (2.26)		7.79 (1.91)	
Temperature, °C^a^	12.47 (5.80)		12.43 (5.80)		12.75 (5.79)	
Glucose, mg/dL^a^	102.02 (36.34)		95.51 (20.06)		145.33 (72.56)	
Cholesterol, mg/dL^a^	194.80 (39.50)		197.14 (38.45)		179.32 (42.73)	

Abbreviations: BMI, body mass index; OR, odds ratio; PM_2.5_, particulate matter with an aerodynamic diameter less than or equal to 2.5 μm; WTC, World Trade Center.

^a^ Values are expressed as mean (standard deviation).

^b^ Calculated as weight (kg)/height (m)^2^.

We observed a 0.78-mg/dL (95% confidence interval (CI): 0.30, 1.26) increase in glucose levels and a 1.67-mg/dL (95% CI: 1.00, 2.35) increase in total cholesterol levels associated with an IQR increase in 6-month average PM_2.5_ exposure. We also observed a 0.75-mg/dL (95% CI 0.26, 1.23) increase in total cholesterol levels associated with an IQR increase in 3-month average PM_2.5_ exposure. We did not find associations between PM_2.5_ exposures averaged 1 week or 3 months before the tests and increased serum glucose (Table 2).

**Table 2:** Estimates^a^ for the Change in Glucose and Total Cholesterol Associated With PM_2.5_ Exposure Averaged 1 Week, 3 Months, or 6 Months Before the Study Visit, World Trade Center Health Program, New York, 2004–2019

**Exposure Window**	**Glucose (*n* = 81,599)**		**Total Cholesterol (*n* = 96,155)**	
	**% Change**	**95% CI**	**% Change**	**95% CI**
1 week	−0.07	−0.33, 0.20	0.32	−0.05, 0.69
3 months	0.11	−0.24, 0.45	0.75	0.26, 1.23
6 months	0.78	0.30, 1.26	1.67	1.00, 2.35

Abbreviations: CI, confidence interval; PM_2.5_, particulate matter with an aerodynamic diameter less than or equal to 2.5 μm.

^a^ We used generalized additive models to estimate the change in glucose and cholesterol levels associated with a 1-unit increase in interquartile range for PM_2.5_ (3.09 μg/m^3^) exposure averaged 1 week, 3 months, or 6 months before the study visit. Separate models were fitted for each outcome. Models adjusted for age, race, educational level, diabetes, hypertension, season, a penalized spline for the year, and average air temperature matching the exposure window of PM_2.5_.

We also present the associations between the WTC-exposure categories and the outcomes adjusted for the 6-month average PM_2.5_ exposure. We observed higher glucose and total cholesterol levels associated with higher WTC exposure categories, treating the lowest exposure category as the reference. The highest exposure category was associated with a 0.82-mg/dL (95% CI: 0.22, 1.43) increase in glucose levels. Additionally, the intermediate category was associated with a 0.99-mg/dL (95% CI: 0.30, 0.1.67) increase in total cholesterol levels (Table 3). Sensitivity analysis restricting the data to samples collected after 2012 supports the same inference, with slightly larger estimates (Web Table 2). Similarly, sensitivity analysis using the hours spent on the WTC site during the clean-up, rescue, and recovery also led to similar inferences (Web Table 3). Finally, when comparing the main models with the abnormally high dichotomous ones, the direction patterns are similar except for the association between high WTC exposure and glucose levels, which did not maintain its significance in the dichotomous model **(**Table 4**)**.

**Table 3:** Estimates^a^ for the Change in Glucose or Total Cholesterol Associated With World Trade Center Exposure Categories, World Trade Center Health Program, New York, 2004–2019

**WTC-Related Exposure Category**	**Glucose (*n* = 82,015)**			**Total Cholesterol (*n* = 96,155)**		
	**No.**	**Unit Change**	**95% CI**	**No.**	**Unit Change**	**95% CI**
Low	11,338	0	Referent	13,929	0	Referent
Intermediate	52,778	0.36	−0.16, 0.88	61,466	0.99	0.30, 1.67
High	17,483	0.82	0.22, 1.43	20,760	0.06	−0.74, 0.86

Abbreviations: CI, confidence interval; PM_2.5_, particulate matter with an aerodynamic diameter less than or equal to 2.5 μm; WTC, World Trade Center.

^a^ We used generalized additive models to estimate the change in glucose and cholesterol levels associated with the WTC-exposure categories. Separate models were fitted for each outcome. Models adjusted for PM_2.5_ exposure averaged six months before the study visit, age, race, education level, diabetes diagnostic, hypertension diagnostic at the time of the testing, season, a penalized spline for the year, and 6-month average air temperature.

**Table 4:** Odds Ratio of Abnormally High Levels of Glucose (>100) and Total Cholesterol (>200) Associated With World Trade Center Exposure Categories and Interquartile Change of 6-Month Average Fine Particulates Exposure, World Trade Center Health Program, New York, 2004–2019^a^

**Exposure**	**Glucose (*n* = 82,015)**			**Total Cholesterol (*n* = 96,155)**		
	**No.**	**OR**	**95% CI**	**No.**	**OR**	**95% CI**
PM_2.5_ 6-month average		1.04	0.99, 1.09		1.08	1.04, 1.12
WTC-related exposure						
Low	11,338	0	Referent	13,929	0	Referent
Intermediate	52,778	1.03	0.98, 1.08	61,466	1.04	1.01, 1.08
High	17,483	1.08	1.02, 1.14	20,760	1.01	0.96, 1.05

Abbreviations: CI, confidence interval; OR, odds ratio; PM_2.5_, particulate matter with an aerodynamic diameter less than or equal to 2.5 μm; WTC, World Trade Center.

^a^ We used generalized additive models to simultaneously estimate the odds ratio of abnormally high levels of glucose (>100 mg/gL) and total cholesterol (>200 mg/dL) associated with 1-unit interquartile-range increase of 6-month averaged PM_2.5_ (3.09 μg/m^3^) and the WTC exposure categories. Models adjusted for age, race, educational level, diabetes, hypertension, season, a penalized spline for the year, and a 6-month average air temperature.

**Figure 1:**
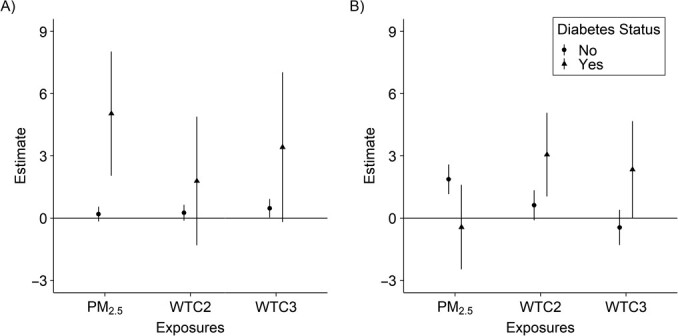
Estimates for the change in glucose (A) and total cholesterol (B) associated with World Trade Center (WTC) exposure categories and interquartile range (IQR) change of 6-month average particulate matter with an aerodynamic diameter less than or equal to 2.5 μm (PM_2.5_) exposure, stratified by diabetes status, World Trade Center Health Program, New York, 2004–2019. We used generalized additive models to estimate the change in glucose and cholesterol levels associated with a 1-unit increase in IQR for PM_2.5_ (3.09 μg/m^3^) and the WTC-exposure categories (reference level: WTC low). PM_2.5_ 6-month average was obtained from a highly resolved spatiotemporally model. WTC exposure incorporates data on total time working at the WTC site, exposure to the cloud of debris from the WTC collapse, and work directly on the pile of debris. Both exposures were included simultaneously in the models. Separate models were fitted for each outcome. Models adjusted for age, race, education level, hypertension diagnostic at the testing time, season, a penalized spline for the year, and 6-month average air temperature. Models were stratified by diabetes status. WTC2, intermediate exposure; WTC3, high exposure.

The associations of WTC exposure and PM_2.5_ with glucose or total cholesterol varied by diabetes status. A 1-IQR increase in 6-month average PM_2.5_ exposure was associated with larger glucose increases among people with diabetes (5.03 mg/dL, 95% CI: 2.04, 8.02) compared with people without diabetes (0.16 mg/dL, 95% CI: –0.19, 0.52). Similarly, we observed higher glucose and cholesterol levels associated with higher WTC-related exposures among those with diabetes. However, the association between PM_2.5_ exposure and total cholesterol was more pronounced among people without diabetes (1.92 mg/dL, 95% CI: 1.21, 2.64) compared with people with diabetes (−0.44 mg/dL, 95% CI: –2.47, 1.58) (Figure 1). Regarding potential reverse causality due to disease status during the WTC attack, our results suggest that neither diabetes nor hypertension diagnostics were associated with WTC-related exposure. Among people with preexisting diabetes (*n* = 456), 15.8%, 62.2%, and 21.9% were exposed to low, intermediate, and high WTC-related exposure levels, respectively. Among people with preexisting hypertension (*n* = 1,598), 16.9%, 59.6%, and 23.5% were exposed to low, intermediate, and high WTC-related exposure levels, respectively. These frequencies do not differ from the distribution of WTC-related exposure among responders who did not have these pre-existing conditions (15.8%, 62.9%, and 21.3% for low, intermediate, and high WTC-related exposure levels, respectively).

Our findings suggest that the mediator role of PM_2.5_ exposure in the association between WTC exposure and both glucose and cholesterol levels is negligible. First, comparing mean PM_2.5_ levels among responders who relocated at least once during the study period, we found similar average PM_2.5_ levels in their first recorded address (8.38 μg/m^3^) and relocation address (8.30 μg/m^3^). Second, the intermediate WTC-related exposure was not associated with PM_2.5_ levels (0.01 μg/m^3^, 95% CI -0.01;0.03). Third, the highest exposure category was associated with a mean decrease of −0.03 μg/m^3^ (95% CI: −0.06, –0.01) in PM_2.5_ levels. In mediation analyses, PM_2.5_ mediated −1.7% (95% CI: −6.2, –1.0) of the association between high WTC exposure and glucose and 33.6% (95% CI: −70.6, 63.0) of the association with cholesterol. Finally, the effect estimates of the models without adjustment for PM_2.5_ were extremely similar to those found in the main analysis (Web Table 4).

## DISCUSSION

Our findings show that extreme air pollution exposure during the WTC disaster and intermediate-term PM_2.5_ exposure are independently associated with increased glucose and total cholesterol levels among WTCHP responders during 20 years of follow-up. The intermediate WTC-exposure category was associated with higher total cholesterol, but the association was attenuated at the highest exposure category. This might be related to residual confounding or exposure measurement error. Finally, people with diabetes were more susceptible to these exposures except for the associations between PM_2.5_ and cholesterol levels.

Systematic reviews have showcased an association between PM_2.5_ and an increase in type 2 diabetes—10-μg/m^3^ increase in PM_2.5_ associated with an odds ratio of 1.09 in meta-analysis (3) and cardiovascular disease risk—approximately 10% risk increase (27). In addition, several studies have found associations between intermediate-term PM_2.5_ exposure and glucose levels (22), cholesterol levels (24), or both (23, 28). For example, a study in Taiwan (23) found a 36.55-mg/dL increase in glucose and 75.95-mg/dL increase in cholesterol per IQR PM_2.5_ change (20.42 mg/dL). A direct comparison with our effect sizes is not possible due to the different methods used to calculate the effect size or the substantial differences in exposure levels. Although the effect estimates observed in our study are small, considering the ubiquitous nature of PM_2.5_ and the extent of the population exposed, these differences are of public health concern. Higher glucose values can be translated into higher cardiovascular disease risk (Shaye et al. (29)) and alter the cardiometabolic risk trajectory even within the normal range.

To our knowledge, our results are the first to investigate and showcase an association between WTC-related exposure and increases in glucose and total cholesterol levels. Previous literature supports an association between non-WTC occupational dust exposure and increased risk of diabetes (30) and diverse cardiovascular disease risk markers (31). Several longitudinal studies have shown associations between WTC-related exposures and heart disease (32), stroke (33), and overall cardiovascular disease risk (34). Others found no association between WTC-related exposure and diabetes or cardiovascular disease (35). Using different WTC exposure measures (i.e., composite indices, day-of-arrival, or total hours of on-site work) could be a potential source for inconsistencies as each measure could implicitly include different information, like trauma or injury. Until now, to our knowledge, diabetes has been associated with WTC exposure only through PTSD (12) and not through dust-related exposures.

A central finding of our study is the higher vulnerability to WTC and PM_2.5_ exposure observed among people with diabetes. We found stronger associations between WTC and PM_2.5_ exposure with glucose and cholesterol levels among people with diabetes, except for the association between PM_2.5_ and cholesterol. Similar to our findings, existing literature has found larger air-pollution health impacts among people with diabetes. For example, Zanobetti et al. (36) found that people with diabetes have a doubled risk of cardiovascular-related hospital admission related to PM_10_ exposure compared with those without diabetes. Similarly, Yitshak-Sade et al. (6) found larger associations of intermediate-term PM_2.5_ exposure on the risk of increased glucose levels among people with diabetes. This increased vulnerability to air pollution, supported by our study, may also be attributed to inflammatory and coagulation process changes and the consequential insulin resistance and vascular dysfunction observed among people with diabetes (37). However, our findings of more substantial associations between PM_2.5_ and total cholesterol in the population without diabetes contradict existing evidence: Yitshak-Sade et al. (6) found stronger associations of PM_2.5_ with low-density lipoprotein and triglycerides among people with diabetes. The stronger PM_2.5_-cholesterol associations among those without diabetes may be due to stratification by other unmeasured variables closely related to diabetes, such as hyperlipidemia and lipid-modifying medications, due to their high correlation with diabetes status.

### Strengths and limitations

The use of the WTCHP is a major strength of this study. First, the participants of WTCHP are thoroughly characterized, which allows for comprehensive adjustment in the models. Second, we could detect environmental health associations with glucose and total cholesterol levels—two critical cardiometabolic health indicators—and capture more subtle changes in the participants’ metabolism. Finally, we observed air pollution’s adverse health associations in an occupational cohort with a relatively healthy population.

However, this study has a few limitations. First, self-reported WTC exposure may lead to potential misclassification. However, previous studies regarding the adverse impacts of WTC-related exposures have used self-reported measures and have found associations between higher exposures and adverse health impacts (18, 38). Similarly, while the composite measure used to capture WTC-related exposure has not been validated, it has been linked to other health outcomes in studies that observed worse health outcomes in higher exposure categories (18–20). Second, an unexposed reference population is unavailable. However, the variability of WTC exposure within the cohort population was sufficient to detect associations with our studied outcomes. In addition, address history was collected only starting in 2012. Therefore, exposures assigned for earlier years may be subject to exposure misclassification. However, our sensitivity analysis suggests that this did not bias the results observed in our study. Third, we did not have access to information on potentially important confounders such as smoking and physical activity. Finally, the selected population included in the WTCHP cohort may limit the generalizability of our findings. However, our study provides evidence of harmful PM_2.5_ associations among a relatively healthy population of workers in which underlying chronic conditions are less prevalent. Additionally, the WTC-related associations observed among this cohort may be supportive of other populations that experienced exposures of a similar nature, such as wildfires and, sadly, future terrorist attacks.

### Conclusion

Our study shows that both extreme air pollution events and intermediate-term PM_2.5_ exposure may have independent metabolic consequences. We observed increases in glucose and total cholesterol levels associated with WTC-related exposure and subsequent intermediate-term residential PM_2.5_ exposure. These observed associations were especially pronounced among people with diabetes. This study adds new evidence on links between WTC exposures and cardiometabolic outcomes. Our findings suggest that extreme air pollution exposure is associated with cardiometabolic health and reaffirm that diabetes status plays an essential role in the association between PM_2.5_ and cardiometabolic health.

Federally funded health care is provided to responders only for diseases certified to be related to 9/11 exposure. However, certifiable diseases are limited to those for which the evidence is robust—mainly cancer and respiratory diseases (39). Cardiometabolic diseases are not currently considered certifiable as the evidence is scarce and mixed (40). Therefore, establishing a new link between WTC exposure and cardiometabolic health might substantially affect health-care costs and accessibility for WTC rescue and recovery workers.

## Supplementary Material

Web_Material_kwad173
