# Supplementary material for: The Association of Air Pollution Exposure With Glucose and Lipid Levels: The Role of an Extreme Air Pollution Event Alongside 2 Decades of Moderate Exposure
Source: Am J Epidemiol. 2023 Aug 16;193(1):87–95. doi: 10.1093/aje/kwad173 (PMC10773474; doi:10.1093/aje/kwad173)
Supplement: Web_Material_kwad173 [file web_material_kwad173.zip › AJE-00914-2022 Knobel Web Material Final.docx]

**Web Material**

**The Association of Air Pollution Exposure With Glucose and Lipid Levels: The Role of an Extreme Air Pollution Event Alongside 2 Decades of Moderate Exposure**

Pablo Knobel ^1^, Allan C. Just ^1^, Elena Colicino ^1^, Susan L. Teitelbaum ^1^, Mary Ann McLaughlin ^2^, Heresh Amini ^3^, Maayan Yitshak Sade ^1^

# ^1^ Icahn School of Medicine at Mount Sinai, Department of Environmental Medicine and Public Health, New York, NY, USA

# ^2^ Division of Cardiology, Department of Medicine, Icahn School of Medicine at Mount Sinai, New York, New York, USA

# ^3^ Department of Public Health, University of Copenhagen, Copenhagen, Denmark

**Table of contents:**

Web Figure 1

Web Tables 1–4

**Web Figure 1:** Directed Acyclic Graph with causal framework.

**
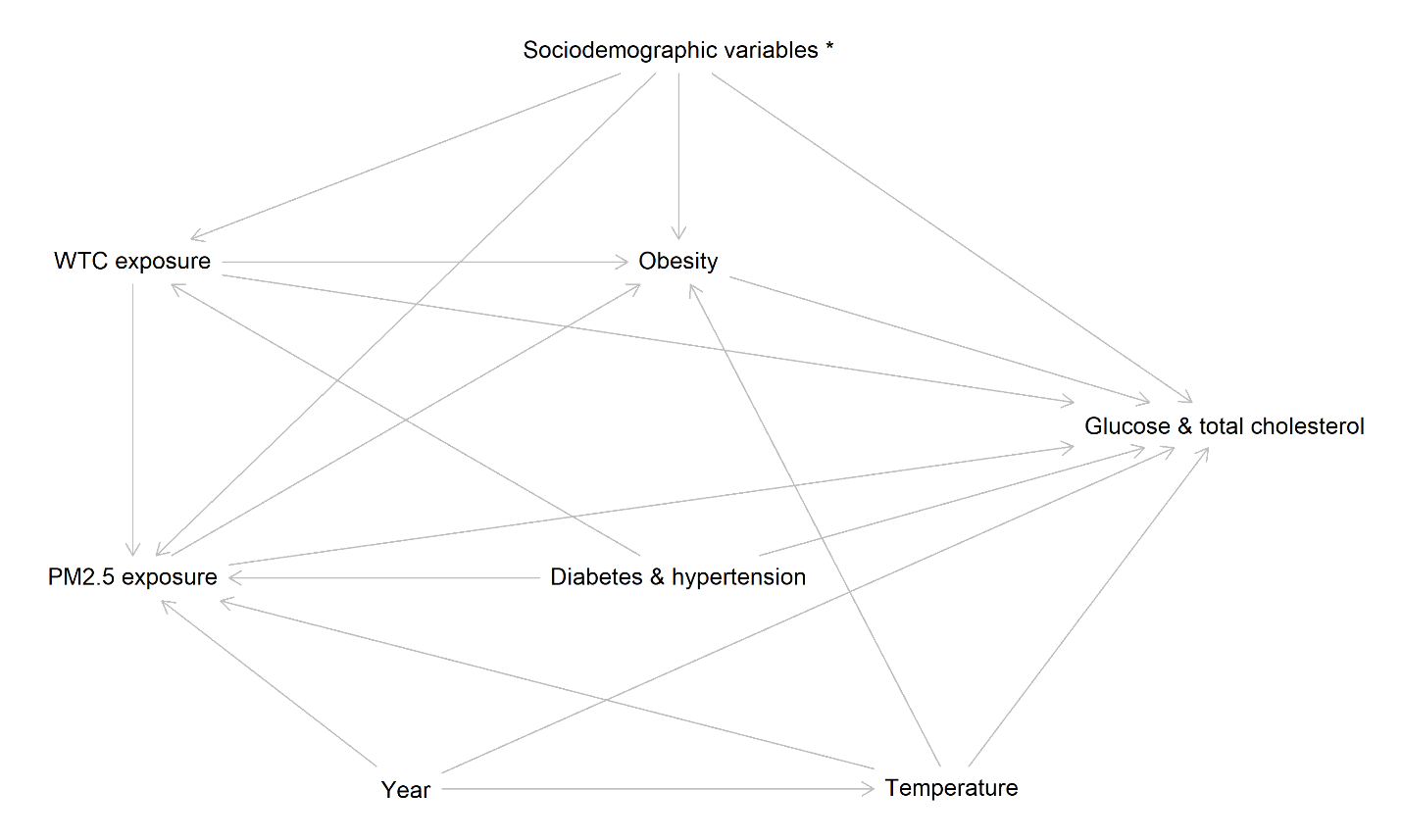
**

* Sociodemographic variables include education level, race, age at visit and sex.

**Web Table 1**: Comparison of population characteristics between blood tests which were included and excluded due to missing data.

| **Variable** | **Included (n=96,155, 87.7%)** | **Excluded ^a^ (n=13,559, 12.3%)** |
| --- | --- | --- |
| Sex, Male n (%) | 81,774 (85.0) | 11751 (86.7) |
| Age at testing (Mean (SD)) | 51.92 (9.33) | 53.19 (9.30) |
| BMI (mean (SD)) | 30.01 (4.94) | 30.32 (5.02) |
| Race n (%) |  |  |
| *Non-Hispanic White* | 60,216 (62.6) | 3026 (52.4) |
| *Non-Hispanic black* | 11,525 (12.0) | 879 (15.2) |
| *Other* | 24,414 (25.4) | 1869 (32.4) |
| *NA* |  | 7785 (57.4) |
| Education level n (%) |  |  |
| *Less than high school* | 7717 ( 8.0) | 573 ( 5.9) |
| *High school* | 20435 (21.3) | 1903 (19.4) |
| *Some college* | 26746 (27.8) | 2965 (30.3) |
| *College or professional school* | 41257 (42.9) | 4348 (44.4) |
| *NA* |  | 3770 (27.8) |
| Diabetes n (%) | 12,644 (13.1) | 1501 (11.1) |
| Hypertension n (%) | 40,897 (42.5) | 4730 (34.9) |
| WTC composite exposure n (%) |  |  |
| *Low* | 13,929 (14.5) | 1938 (18.4) |
| *Intermediate* | 61,466 (63.9) | 6577 (62.3) |
| *High* | 20,760 (21.6) | 2036 (19.3) |
| *NA* |  | 3008 (22.2) |
| PM_2.5_, μg/m^3^ (Mean (SD)) | 8.24 (2.23) | 7.55 (1.88) |
| Temperature, Cº (Mean (SD)) | 12.47 (5.80) | 12.26 (5.75) |
| Glucose (Mean (SD)) | 102.02 (36.34) | 101.65 (31.22) |
| Cholesterol (Mean (SD)) | 194.80 (39.50) | 192.43 (38.35) |

SD=standard deviation

**^a^** percentages were calculated out of non-missing data. Percent missing (NA) is also presented.

**Web Table 2:** Sensitivity analysis - Estimates for the change in glucose and total cholesterol associated with WTC exposure groups for a restricted (2012-2019) study period.

|  | **WTC-Exposure** | **Unit change (95% CI)** | |
| --- | --- | --- | --- |
| **Sample** |  | **Glucose (n = 44,233)** | **Total** **Cholesterol (n = 55,039)** |
| 2012-2019 | Low |  |  |
|  | Intermediate | 0.24 ( -0.49 ; 0.97 ) | 1.4 ( 0.52 ; 2.29 ) |
|  | High | 0.95 ( 0.1 ; 1.8 ) | 0.65 ( -0.38 ; 1.68 ) |

We used generalized additive models to estimate the change in glucose and cholesterol levels associated with the WTC-exposure categories. Models were adjusted for age, race, education level, diabetes, hypertension, season, a penalized spline fo the year, and 6-month average PM_2.5_ and air temperature. Sample size of each WTC exposure group: glucose [low: 6,290 , intermediate: 28,369, high: 9,574] ;cholesterol [low: 8,225, intermediate: 34,797, high:11,990].

**Web Table 3:** Sensitivity analysis - Estimates for the change in glucose and total cholesterol associated with a 10 hour increase in time spent on the WTC site during the clean-up, rescue, and recovery.

| **WTC-Exposure** | **Unit change (95% CI)** | |
| --- | --- | --- |
|  | **Glucose (n= 81,483‬)** | **Total** **Cholesterol (n = 95,997)** |
| Hours spent on site | 0.03 ( 0.01 ; 0.04 ) | 0.01 ( -0.01 ; 0.04 ) |

We used generalized additive models to estimate the change in glucose and cholesterol levels associated with a 10 hour increase in time spent on the WTC site during the clean-up, rescue, and recovery. Models were adjusted for age, race, education level, diabetes, hypertension, season, a penalized spline fo the year, and 6-month average PM_2.5_ and air temperature.

**Web Table 4:** Mediation analysis - Estimates for the change in glucose or total cholesterol associated with WTC exposure categories without adjustement for PM_2.5_.

| **Exposure** | **Unit change (95% CI)** | |
| --- | --- | --- |
|  | Glucose (N=82,015) | Total Cholesterol (N=96,155) |
| WTC-Related exposure  Low | Reference group | Reference group |
| Intermediate | 0.36 ( -0.16 ; 0.88 ) | 0.99 ( 0.31 ; 1.68 ) |
| High | 0.81 ( 0.2 ; 1.42 ) | 0.05 ( -0.76 ; 0.85 ) |

We used generalized additive models to estimate the change in glucose and cholesterol levels associated with the WTC-exposure categories. Separate models were fit for each outcome. Models were adjusted age, race, education level, diabetes diagnostic, season, a penalized spline fo the year, and 6-month average air temperature. Sample size of each WTC exposure group: glucose [low: 11338, intermediate: 52778, high: 17483] ;cholesterol [low: 13929, intermediate: 61466, high: 20760].
